# Gender-dependent resiliency to stressful and metabolic challenges following prenatal exposure to high-fat diet in the p66^Shc−/−^ mouse

**DOI:** 10.3389/fnbeh.2014.00285

**Published:** 2014-08-22

**Authors:** Veronica Bellisario, Alessandra Berry, Sara Capoccia, Carla Raggi, Pamela Panetta, Igor Branchi, Giovanni Piccaro, Marco Giorgio, Pier G. Pelicci, Francesca Cirulli

**Affiliations:** ^1^Section of Behavioral Neurosciences, Department of Cell Biology and Neurosciences, Istituto Superiore di SanitàRome, Italy; ^2^Section of Bacterial, Respiratory and Systemic Diseases, Department of Infectious, Parasitic and Immune-Mediated Diseases, Istituto Superiore di SanitàRome, Italy; ^3^Department of Experimental Oncology, European Institute of OncologyMilan, Italy

**Keywords:** maternal obesity, p66^Shc^ gene, oxidative stress, biomarkers, adipokines, gender, emotionality, animal models

## Abstract

Metabolic stressful challenges during susceptible time windows, such as fetal life, can have important implications for health throughout life. Deletion of the p66^Shc^ gene in mice leads to reduced oxidative stress (OS), resulting in a healthy and lean phenotype characterized by increased metabolic rate, resistance to high-fat diet (HFD)-induced obesity and reduced emotionality at adulthood. Here we hypothesize that p66^Shc−/−^ (KO) adult offspring might be protected from the detrimental effects induced by maternal HFD administered before and during pregnancy. To test such hypothesis, we fed p66^Shc+/+^ (WT) and KO females with HFD for 13 weeks starting on 5 weeks of age until delivery and tested adult male and female offspring for their metabolic, neuroendocrine, and emotional profile. Prenatal diet affected stress responses and metabolic features in a gender-dependent fashion. In particular, prenatal HFD increased plasma leptin levels and decreased anxiety-like behavior in females, while increasing body weight, particularly in KO subjects. KO mice were overall characterized by metabolic resiliency, showing a blunted change in glycemia levels in response to glucose or insulin challenges. However, in p66^Shc−/−^ mice, prenatal HFD affected glucose tolerance response in an opposite manner in the two genders, overriding the resilience in males and exacerbating it in females. Finally, KO females were protected from the disrupting effect of prenatal HFD on neuroendocrine response. These findings indicate that prenatal HFD alters the emotional profile and metabolic functionality of the adult individual in a gender-dependent fashion and suggest that exposure to high-caloric food during fetal life is a stressful condition interfering with the developmental programming of the adult phenotype. Deletion of the p66^Shc^ gene attenuates such effects, acting as a protective factor.

## Introduction

The environment experienced early during development is not only crucial for setting the growth trajectory of the fetus but represents also a key factor contributing to overall disease susceptibility in later life (Barker, 1995). In this context, developmental plasticity is a fundamental mechanism matching the growing organism to the environment it will face after birth (Barker, 2003). Early life stress can deeply influence developmental programming. In modern society maternal obesity—often associated with low socio-economic status—is an example of a physiological stressor experienced by women during pregnancy (Reynolds et al., 2013). Preclinical evidence, recently confirmed by clinical studies, suggest that such metabolic condition produces an adverse *in utero* environment for the offspring with long-term detrimental effects (Drake and Reynolds, 2010; Li et al., 2011). In particular, maternal obesity induces a chronic mild inflammatory state and high levels of oxidative stress (OS), resulting in frailty for psychiatric disorders and metabolic complications linked to an increased risk of insulin resistance (IR) and type 2 diabetes (T2D), in addition to cardiovascular disease and other metabolic disorders associated with obesity (Kahn and Flier, 2000; Wang et al., 2004; Fantuzzi, 2005; Taylor et al., 2006; Eriksson et al., 2014). Likewise, high-fat feeding during pregnancy, independently from maternal obesity, causes *per se* placental dysfunction and metabolic impairment in the offspring (McCurdy et al., 2009; Frias et al., 2011). Preclinical studies have shown that extreme changes in maternal diet influence maternal stress responses and, likewise, affect offspring outcome, including adverse changes in behavior and memory (Weinstock, 2001), in cardiovascular responses to stress (Igosheva et al., 2007), in glucose tolerance (Lesage et al., 2004), as well as sexual dimorphisms of brain regions associated with mood and hypothalamic-pituitary-adrenal (HPA) axis functions (Handa et al., 1994; Majdic and Tobet, 2011). Analyzing further these mechanisms could help identifying protective factors and lead to a better understanding of sex differences in the risk for metabolic and mood disorders reported in the adult population. These are important public health issues, given the abundance of dietary fats in western diets.

The p66^Shc^ gene is a mammalian gerontogene involved in metabolism and OS (Trinei et al., 2009) and plays a major role in the aging process. p66^Shc^ is highly expressed within the adipose tissue and is involved in adipogenesis as it contributes to the intracellular insulin-mediated signaling pathway regulating fat accumulation (Berniakovich et al., 2008; Tomilov et al., 2011). In addition to resistance to OS, the lack of p66^Shc^ gene leads to reduced trygliceride accumulation in the adipocytes, reduced fat mass, increased metabolic rate and resistance to diet-induced obesity in the mouse (Berniakovich et al., 2008; Tomilov et al., 2011). Furthermore, the p66^Shc^ mutants show reduced emotionality, both in social and non-social contexts (Berry et al., 2007, 2008; Berry and Cirulli, 2013), associated to a mild hyperdrive of the HPA axis (Berry et al., 2010). These physiological features of the p66^Shc−/−^ mouse make it a powerful tool to investigate the mechanisms underlying the long-term consequences of maternal exposure to an obesogenic diet on metabolism and emotionality of the adult offspring.

The current study was aimed at investigating the long-lasting effects of maternal high-fat feeding on the neuroendocrine, metabolic and behavioral phenotype of the adult offspring, paying particular attention to gender-specific outcomes. In addition, we aimed at identifying individual metabolic and/or neuroendocrine markers of vulnerability to metabolic and emotional disorders.

Given the role played by the p66^Shc^ gene in cellular metabolism, by mediating the insulin signaling, we hypothesized that the lack of this gene might exerts a protective role against the detrimental effects of maternal exposure to high-fat diet (HFD). To test such hypothesis, p66^Shc+/+^ (WT) and p66^Shc−/−^ (KO) female mice were fed with HFD or control diet (CD) for 13 weeks: from 5 weeks of age until right before delivery. Indeed, while several studies on the effects of maternal high-fat feeding have been performed by extending HFD exposure through pregnancy and/or lactation up until weaning, in this study we wanted to focus mainly on prenatal effects. The offspring was subsequently phenotyped for metabolic, neuroendocrine and behavioral responses.

## Materials and methods

### Animals

Experimental subjects were 5 weeks-old knock-out (KO—p66^Shc−/−^, *n* = 71) and wild-type (WT—p66^Shc+/+^, *n* = 77) female mice on a C57BL6/J background. Animals were housed 2/cage in transparent Plexiglas cages (37 × 21 × 19 cm) provided by Tecniplast, in an air conditioned room (temperature 21 ± 1°C, relative humidity 60 ± 10%) under a reversed 12/12 h light/dark cycle with lights off from 07:00 a.m. to 07:00 p.m. Fresh tap-water and standard chow (standard diet -SD- energy 3.3 kcal/g, fat 17%, carbohydrate 60%, and protein 23%, provided by Altromin-R, Rieper, Italy) were continuously available until 5 weeks of age. Thereafter females were fed *ad libitum* either with HFD (energy 5.56 kcal/g, fat 58%, carbohydrate 25.5% and protein 16.4%; *n* = 40 WT; 37 KO) or control diet (CD- energy 4.07 kcal/g, fat 10.5%, carbohydrate 73.1% and protein 16.4%; *n* = 37 WT; 34 KO) for 10 weeks, i.e. until 15 weeks of age. Females of both genotypes were randomly assigned to HFD or CD groups avoiding difference in the average of body weight between groups. HFD (D12331) and CD (D12328) were provided by Research Diets, Inc., New Brunswick, NJ, USA. After 10 weeks on the diet, all females were mated with males of the same genotype. Body weight was monitored once a week to assess pregnancy. Pregnant females (*n* = 24 WT-CD; 19 KO-CD; 34 WT-HFD; 27 KO-HFD) were kept with either HFD or CD throughout gestation until 3 days before the expected delivery date, i.e. at gestational day 16 (G16). Thereafter all of them were switched to SD during lactation until pups weaning, occurring at post-natal day 30 (P30). Daily food consumption (24 h) and body weight gain of females maintained on HFD or CD were monitored once a week. The success of birth and the maternal behavior immediately after the birth of pups were also registered. In addition to nest dimension, pups' body weight was also monitored during development at P3, P30 and at 3-months-old (P90). At weaning all pups were weaned onto SD and the onset of puberty markers were checked daily. At 3 months of age, both males and females offspring were tested to assess the metabolic, neuroendocrine and emotional profiles resulting from prenatal exposure to a hypercaloric diet. At this age the Body Mass Index (BMI) of all subjects was also calculated as the ratio between body weight (g) and the square of the anal-nasal length (cm).

**Figure 1:**
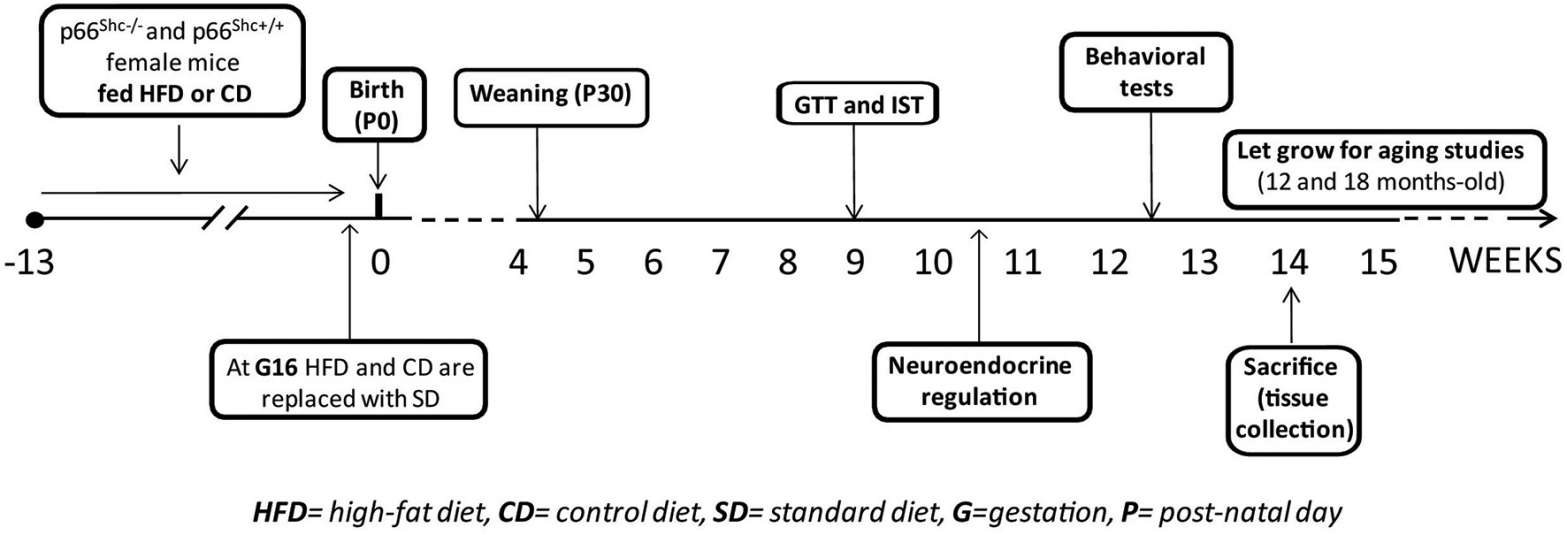
**Schematic design of the experimental plan**.

A schematic design of the experimental plan is reported in Figure 1.

Animal handling and experimental procedures were performed in accordance with the EC guidelines (EC Council Directive 86/609 1987) and with the Italian legislation on animal experimentation (Decreto L.vo 116/92).

### Experimental procedure

#### Onset of puberty

The onset of puberty was assessed in all male and female pups, starting from the day of weaning (P30). Vaginal opening (VO) and balano-preputial separation (BPS) were chosen as pubertal markers (Korenbrot et al., 1977; Rodriguez et al., 1997). Beginning on the day of weaning, the dates of VO for females and BPS for males were recorded. In female mice, VO was determined by daily inspection and recorded as the day on which the vaginal orifice transitioned from tightly closed to patent (Nelson et al., 1990; Azooz et al., 2001; Zhou et al., 2007; Brill and Moenter, 2009). The opening of the vaginal cavity to the skin is an event occurring around the fifth week of life that can be induced in sexually immature mice by beta-estradiol injections (Rodriguez et al., 1997). The separation of the prepuce from the gland penis (balanus) has been shown to be androgen dependent and to occur around the time of puberty (Korenbrot et al., 1977).

#### Metabolic regulation

- **Glucose tolerance test (GTT)**. Intra-peritoneal GTT was performed after a 15 h overnight fasting that took place from 06:30 p.m. until 09:30 a.m. Animals were intra-peritoneally (IP) loaded with 2 g/kg body weight D-glucose (10% D glucose solution; Sigma, St. Louis, MO, USA) (Satapathy et al., 2011). Blood was collected from the tail vein at 0 (baseline), 30, 60, 120 and 180 min (Ranieri et al., 2010) following IP injection and glycemia (blood glucose concentration) was measured using a commercial glucometer (StatStrip Xpress-i, nova biomedical, A. Menarini diagnostic) (Titta et al., 2010).
- **Insulin sensitivity test (IST)**. The test was performed on animals starved for 5 h that took place from 09:30 a.m. until 02:30 p.m. Glycemia was measured using a commercial glucometer (StatStrip Xpress-i, nova biomedical, A. Menarini diagnostic) immediately before (0) and 15, 30, 60, 120 min after IP injection of a 0.4 U/kg body weight (Titta et al., 2010) solution of human recombinant insulin (Humulin, Eli-Lilly, 100 U/mL) (Ranieri et al., 2010).
- **Metabolic hormones assessment**. Plasma levels of leptin and adiponectin were assessed under starving condition. Blood samples were collected at 08:30 a.m., 12 h after removal of the food, from the tail vein in potassium EDTA coated tubes (1.6 mg EDTA/ml blood; Sarstedt, Germany). After centrifugation, plasma samples were used for the determination of leptin levels, by a Mouse Leptin Elisa kit (Crystal Chem Inc., Downers Grove, IL) (Berniakovich et al., 2008), in addition to adiponectin levels by an Elisa kit (B-Bridge International, Inc.) (Giorgio et al., 2012). The levels of adiponectin were also assessed in the epididymal/periovaric and mesenteric adipose tissues by immunoblot analysis. Epididymal/periovaric and mesenteric adipose tissue pads (100 mg) were homogenized with Politron homogenizer in NaCl 150 mM, Tris 25 mM pH 6.8, EDTA 1 mM, supplemented with protease and phosphatase inhibitors, and centrifugated 1000 g for 10 min at 4°C, the fat pad fraction was discarded. A Bradford protein assay (Bio-Rad) was then conducted on the samples to determine the protein concentration for each sample. Proteins (20 mg) were separated by SDS-PAGE (10%) and transferred to nitrocellulose membranes. Membranes were blocked in 5% non-fat dry milk for 1 h and incubated with primary antibody for 1 h. Membranes were washed with TBS/0.1% Tween-20 three times and incubated with secondary antibody for 1 h, then washed with TBS/0.1% Tween-20 three times and reactivity was detected by the enhanced chemiluminescence kit (Pierce). Blots were developed using a FluorChem™ R System. Mouse monoclonal adiponectin and actin antibodies were from Abcam, Inc. (Cambridge, MA).

#### Neuroendocrine activation

The activation of the HPA axis was assessed in response to a psychophysical stressful challenge. All subjects underwent an acute restraint stress (30 min) and blood samples were collected by a tail nick at different time points, i.e. soon before (0 min) and following (30, 180 and 240 min) the exposure to stress, in order to measure plasma levels of corticosterone (CORT) (MP Biomedicals, LLC, Germany GmbH). Exposure to stress took place at 02:30 p.m., when the levels of free CORT were far from the circadian peak (Kitchener et al., 2004).

#### Behavioral phenotype

After 10 days of washout from the acute restraint stress, all subjects underwent behavioral testing to assess spontaneous behavior in the Open Field test, in addition to emotionality and general anxiety in the Elevated Plus Maze test (Pellow et al., 1985; File, 1993). These two tests were performed on different days.

***Open field (OF)***. Each subject was individually placed in the center of a cubic arena (open field box 40 × 40 × 40 cm) made of Plexiglas and allowed to freely explore for a single session lasting 15 min. The OF box was ideally divided into 25 squares and ideally partitioned into a central portion (24 × 24 cm) and a peripheral one, identified as the remaining part of the arena. When data were analyzed, the session was subdivided in three time blocks (tb), lasting 5 min, and the time spent in each portion of the arena was measured. Furthermore, the duration of locomotion (*crossings* of squares limits with all paws) was scored as index of exploratory behavior.

***Elevated plus maze (EPM)***. The EPM is made of two open arms (30 × 5 × 0 cm) and two closed arms (30 × 5 × 15 cm) that extend from a common central platform (5 × 5 cm). The apparatus, made of Plexiglas (gray floor, clear walls), is elevated to a height of 60 cm above the floor. Mice were individually placed on the central platform facing an open arm and allowed to freely explore the maze for 5 min. Behavioral parameters observed were: % open entries [(open/total) × 100] and time spent in the open and closed arms of the maze (File, 2001). Furthermore, the behavioral parameters taken into account were the total amount of time spent in *immobility* and *self-grooming*, a self-directed behavior providing a reliable marker of anxiety (Kalueff and Tuohimaa, 2005).

In all behavioral tests, after each trial/session, the apparatus was cleaned with 50% ethanol to reduce olfactory cues.

At 9 weeks of age mice began the experimental procedure. All subjects underwent the GTT as first metabolic test and after 3 days, in which mice were left undisturbed, all of them were tested for the IST. This time interval was necessary to recover from the fasting and the handling procedures. Five days after the IST the neuroendocrine activation was assessed. After 10 days from the end of the stress procedure subjects underwent the behavioral tests.

#### Tissue collection

At 3 months of age all subjects were sacrificed and the epididymal/periovaric and mesenteric adipose tissue depots dissected out and immediately stored at −80°C for further analysis.

### Statistical analysis

Data were analyzed using parametric analysis of variance (ANOVA) with diet (HFD vs. CD), genotype (WT vs. KO) and gender (females vs. males) as between-subjects factors and minutes, zones (EPM: “center” vs. “closed arms” vs. “open arms”; OF: “center” vs. “periphery”) and time blocks (0–5 vs. 5–10 vs. 10–15 min) as within-subject repeated measures, when appropriate (GTT, IST, CORT measurement, EPM and OF tests). *Post-hoc* comparisons have been performed using the Tukey's test. A linear regression model was used to assess the effect of body weight on the onset of puberty in the offspring. Fisher's exact probability test was used to compare genotypes and diets for reproductive success of the colony (i.e. number of pregnant females died during the perinatal period and number of litters in which cannibalistic episodes took place: two-by-two contingency table). Statistics were performed with Statview II (Abacus Concepts, CA, USA).

## Results

### Dams' body weight and food consumption

**Figure 2:**
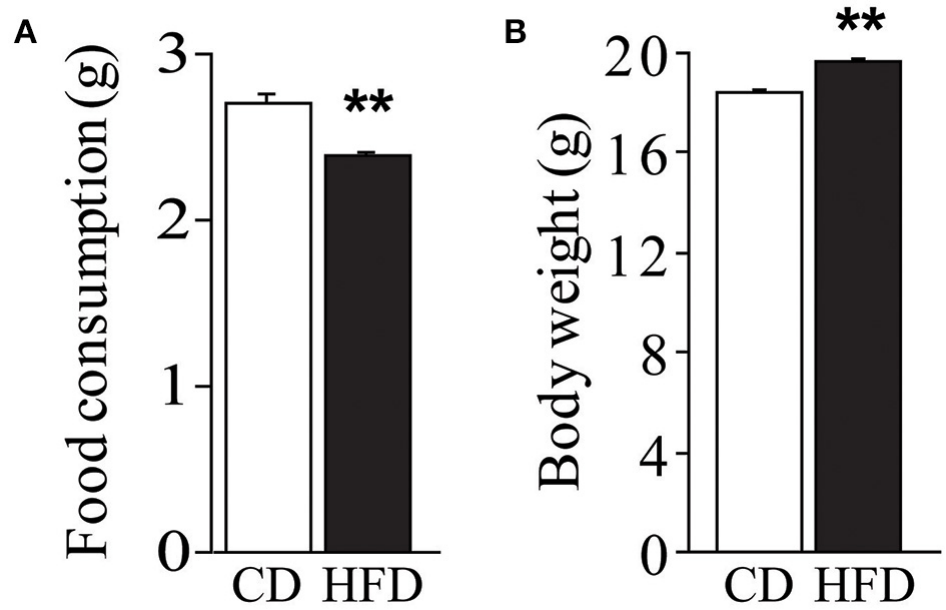
**Effect of high-fat feeding on dams**. Food consumption **(A)** and body weight **(B)** of dams feed HFD. Data are shown as +s.e.m. *Post-hoc* comparisons, ^**^p < 0.01 (hfd vs. cd). Experimental subjects, cd-wt/ko, *n* = 37/34; hfd-wt/ko, *n* = 40/37.

Regardless of genotype, dams fed HFD registered a lower daily food consumption than controls [main effect of diet: *F*_(1, 59)_ = 14.023, *p* = 0.0004, Figure 2A]. Nevertheless, regardless of genotype, HFD determined an increase in body weight during the 10 weeks on diet [main effect of diet: *F*_(1, 144)_ = 22.348, *p* < 0.0001, Figure 2B].

### Success of birth, cannibalistic behavior and litter size

Feeding HFD results in an increased dams' mortality associated to pregnancy (Fisher's exact probability test: *p* = 0.0266, Table 1A) and increased frequency of cannibalistic episodes (Fisher's exact probability test: *p* = 0.0308, Table 1B) only in WT subjects; interestingly such phenomena were strongly reduced in the p66^Shc−/−^ dams. Regardless of genotype, the HFD affected the litter size reducing the number of pups [main effect of maternal diet: *F*_(1, 64)_ = 9.134, *p* = 0.0036, Table 1C].

**Table 1:** **Dams mortality and cannibalistic behavior associated to pregnancy**.

**Success of birth, cannibalistic behavior and pups per litter**			
**(A) DAMS'MORTALITY**			
**Diet**	**Genotype**	**Dead**	**Alive**
CD	p66^Shc+/+^	8	16
CD	p66^Shc−/−^	3	16
HFD	p66^Shc+/+^	11	23
HFD	p66^Shc−/−^	2	25
**(B) DAMS' CANNIBALISTIC BEHAVIOR**			
**Diet**	**Genotype**	**Cannibalism**	**No cannibalism**
CD	p66^Shc+/+^	5	16
CD	p66^Shc−/−^	3	16
HFD	p66^Shc+/+^	8	23
HFD	p66^Shc−/−^	1	25
**(C) NUMBER OF PUPS PER LITTER**			
**Diet**	**Genotype**	**Mean**	
CD	p66^Shc+/+^	5.3 ± 2.4	
CD	p66^Shc−/−^	4.5 ± 2.1	
HFD	p66^Shc+/+^	3.3 ± 2.2	
HFD	p66^Shc−/−^	3.3 ± 1.8	

*Reduced mortality associated to pregnancy **(A)** and reduced frequency of cannibalistic episodes **(B)** in KO females fed HFD. Reduced size of litters on HFD **(C)**. In the section **(A,B)**, the table reports the number of dams on diet; in the section **(C)** the table reports the average number of pups per litter ± s.e.m*.

### Offspring body weight and BMI

**Figure 3:**
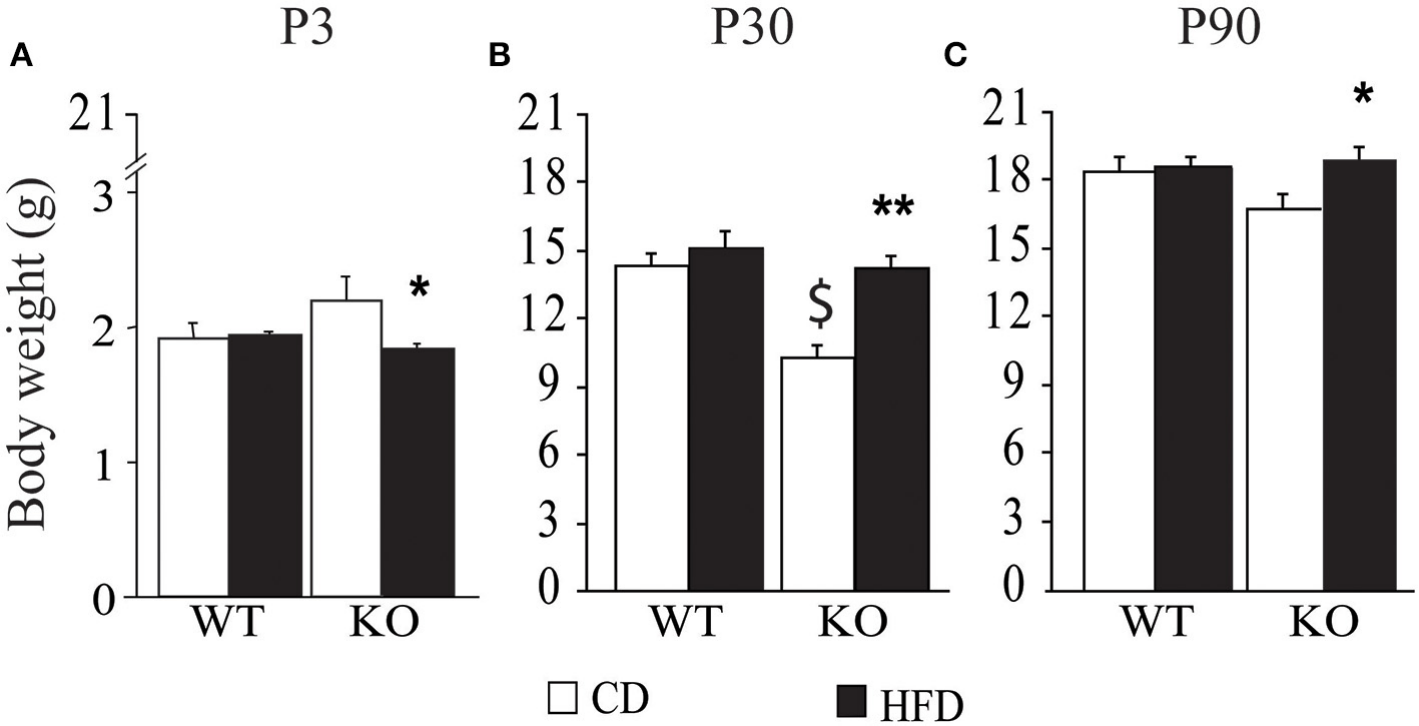
**Body weight of the offspring after birth, P3 (A), at weaning, P30 (B), and at 3 months of age, P90 (C)**. Data are shown as +s.e.m. *Post-hoc* comparisons, ^*^p < 0.05 (P3 and P90: hfd-ko vs. cd-ko); ^$^*p* < 0.01 (P30: cd-ko vs. cd-wt); ^**^p < 0.01 (P30: hfd-ko vs. cd-ko). Experimental subjects, P3: cd-wt, *n* = 16; cd-ko, *n* = 15; hfd-wt, *n* = 15; hfd-ko, *n* = 20; P30 and P90: cd-wt-f/m *n* = 15/10; cd-ko-f/m *n* = 9/14; hfd-wt-f/m *n* = 11/9; hfd-ko-f/m *n* = 11/14.

**Figure 4:**
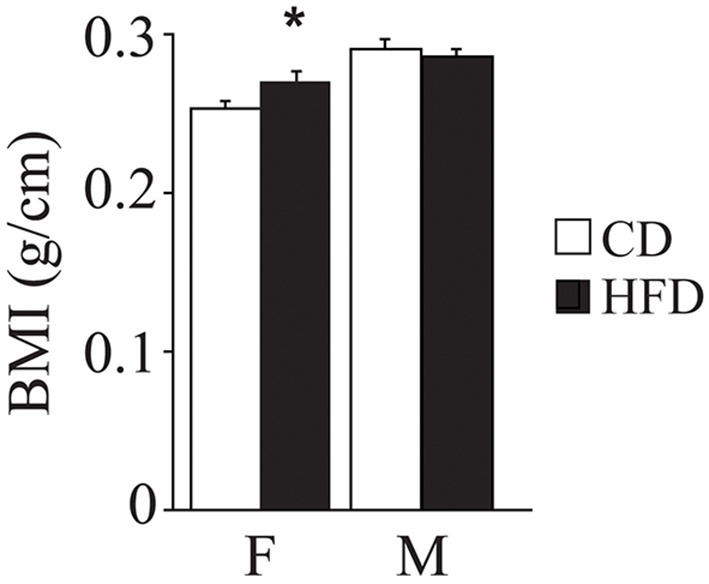
**Body mass index**. Data are shown +s.e.m. *Post-hoc* comparisons, ^*^p < 0.05 (hfd-f vs. cd-f). Experimental subjects, cd-wt-f/m *n* = 8/9; cd-ko-f/m *n* = 8/9; hfd-wt-f/m *n* = 8/9; hfd-ko-f/m *n* = 7/10.

Overall, exposure to maternal HFD resulted in a decreased body weight at P3 [main effect of maternal diet: *F*_(1, 58)_ = 3.835, *p* = 0.05, data not shown], in the p66^Shc−/−^ offspring [interaction between maternal diet and genotype: *F*_(1, 58)_ = 12.120, *p* = 0.0010, Figure 3A]. This effect was reverted during growth (P30) [main effect of maternal diet: *F*_(1, 85)_ = 15.225, *p* = 0.0002, data not shown], particularly in males [interaction between maternal diet and gender: *F*_(1, 85)_ = 4.099, *p* = 0.0461, data not shown]. In addition, at this age, a specific HFD-induced increase in body weight was found in the p66^Shc−/−^ offspring [interaction between maternal diet and genotype: *F*_(1, 85)_ = 6.252, *p* = 0.0143, Figure 3B]. Thus, prenatal HFD was able to override the p66^Shc−/−^ lean phenotype (Figure 3B). Overall, the fatting effect of the HFD was maintained until adulthood (P90), when HFD offspring, regardless of genotype and gender, showed a higher body weight than CD subjects [main effect of maternal diet: *F*_(1, 85)_ = 4.930, *p* = 0.0291, data not shown]. The interaction between maternal diet and gender, observed at P30, was overturned during growth (P90), when higher body weight was observed only in HFD females [interaction between maternal diet and gender: *F*_(1, 85)_ = 6.018, *p* = 0.0162, data not shown]. As for P30, while no difference was observed in WT subjects, KO-HFD offspring maintained a higher body weight compared to KO-CD until 3 months of age (P90) [interaction between maternal diet and genotype: *F*_(1, 85)_ = 8.236, *p* = 0.0052, Figure 3C], particularly in males [interaction among maternal diet, genotype and gender: *F*_(1, 85)_ = 8.447, *p* = 0.0047, data not shown]. Regardless of genotype, maternal HFD reduced the difference in BMI usually observed between genders, increasing the BMI of female mice and rendering them more similar to males [interaction between maternal diet and gender: *F*_(1, 60)_ = 3.820; *p* = 0.05, Figure 4].

### Onset of puberty

**Figure 5:**
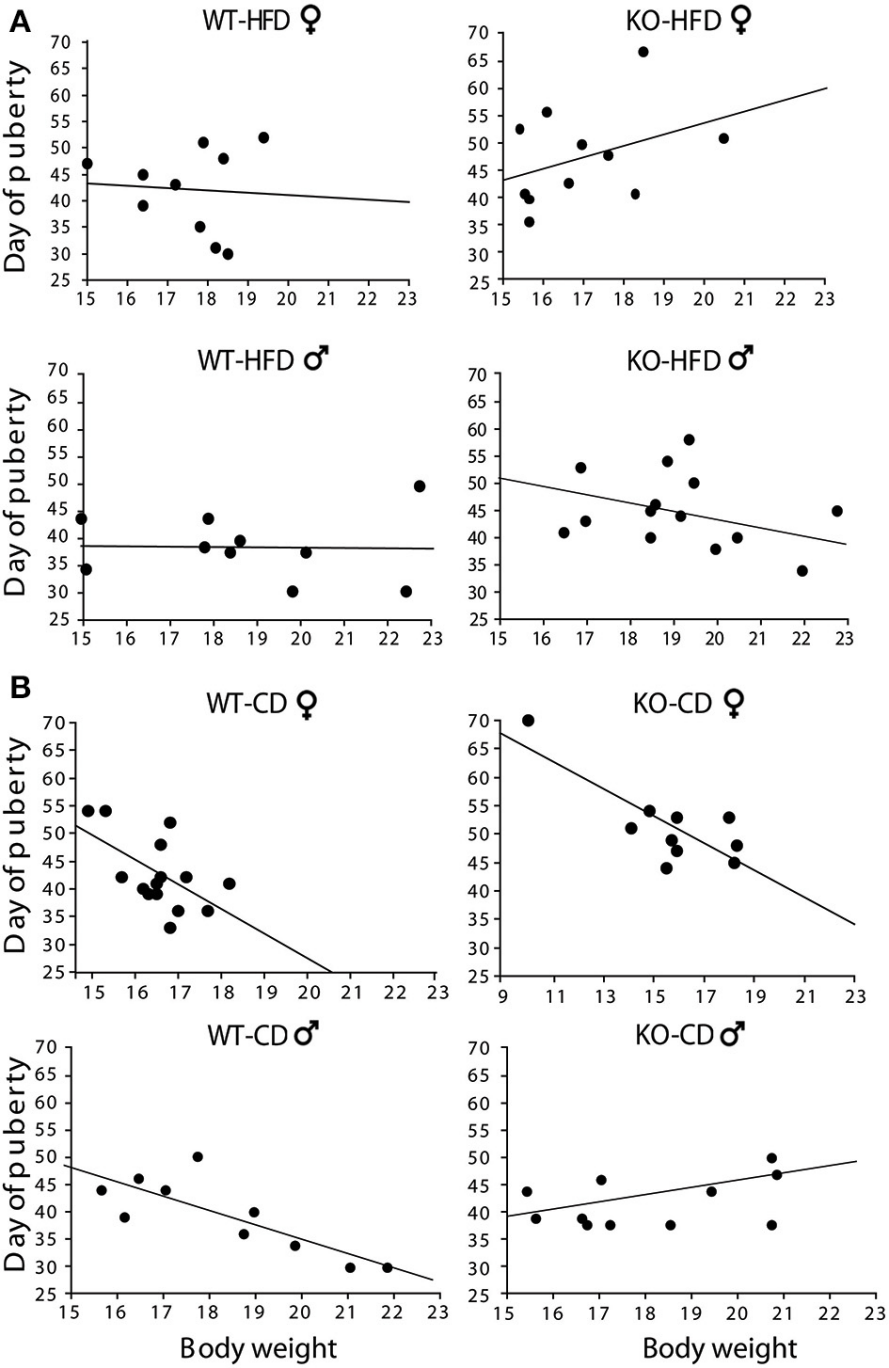
**Onset of puberty**. Correlation between offspring's body weight and timing of puberty in male and female HFD **(A)** and CD **(B)** offspring. Experimental subjects: cd-wt-f/m, *n* = 15/10; cd-ko-f/m, *n* = 10/11 hfd-wt-f/m, *n* = 10; hfd-ko-f/m, *n* = 11/14.

Offspring of HFD- and CD-fed dams did not differ as for their onset of puberty [main effect of maternal diet: *F*_(1, 130)_ = 0.750; *p* = 0.3881; interaction among maternal diet, genotype and gender: *F*_(1, 130)_ = 0.790; *p* = 0.3757, data not shown]. However, and most intriguingly, while in CD subjects this parameter was associated to changes in body weight [*F*_(1, 14)(1, 9)(1, 9)(1, 10)_ = 6.213; 12.808; 14.582; 6.526, *p* = 0.027; 0.0072; 0.0051; 0.0339, *R*^2^ = 0.323; 0.616; 0.646; 0.449, respectively for females WT-CD and KO-CD; males WT-CD and KO-CD, Figure 5B], this did not occur in HFD offspring [*F*_(1, 19)(1, 10)(1, 19)(1, 13)_ = 0.041; 1.496; 0.006; 0.923, *p* = 0.8945; 0.8444; 0.9419; 0.3557, *R*^2^ = 0.005; 0.143; 0.001; 0.071, respectively for females WT-HFD and KO-HFD; males WT-HFD and KO-HFD, Figure 5A], suggesting that the exposure to HFD during fetal programming might have affected the surge of gonadal hormones, resulting in a disorganized pubertal development. Regardless of diet, lack of p66^Shc^ delayed the time of puberty [main effect of genotype: *F*_(1, 130)_ = 6.987, *p* = 0.0092, data not shown] probably by the lower body weight characterizing the transgenic model and that is known to be relevant in the onset of puberty. This latter point could also justify the delayed onset of puberty observed in female mice [main effect of gender: *F*_(1, 130)_ = 20.935, *p* < 0.0001, data not shown].

### Metabolic regulation

**Figure 6:**
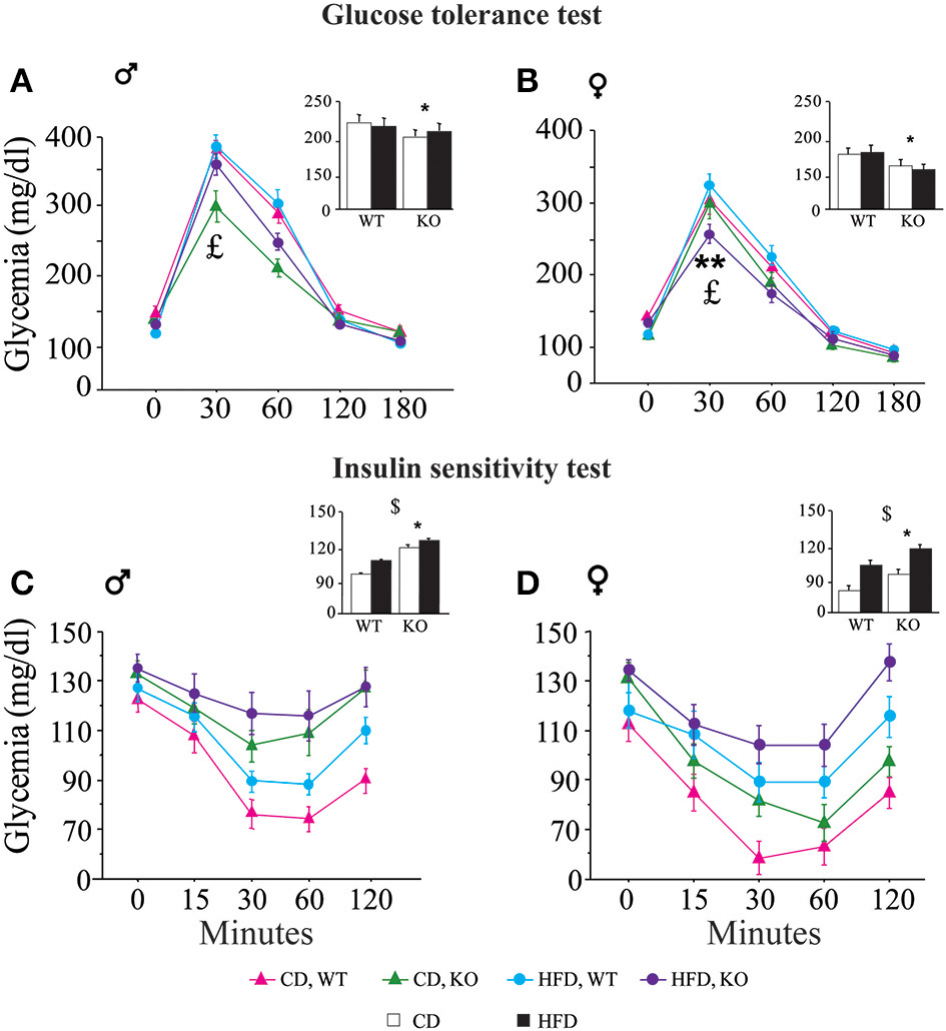
**Metabolic regulation**. Glucose tolerance assessment in WT and KO male **(A)** and female **(B)** offspring fed CD or HFD. Insulin sensitivity test in WT and KO male **(C)** and female **(D)** offspring fed CD or HFD. Data are shown as ±s.e.m. *Post-hoc* comparisons, ^$^*p* < 0.05 (cd vs. hfd); ^*^p < 0.05 (ko vs. wt); ^**^p < 0.01. Experimental subjects: cd-wt-f/m, *n* = 17/22; cd-ko-f/m, *n* = 18/19; hfd-wt-f/m, *n* = 18/22; hfd-ko-f/m, *n* = 17/18.

**Figure 7:**
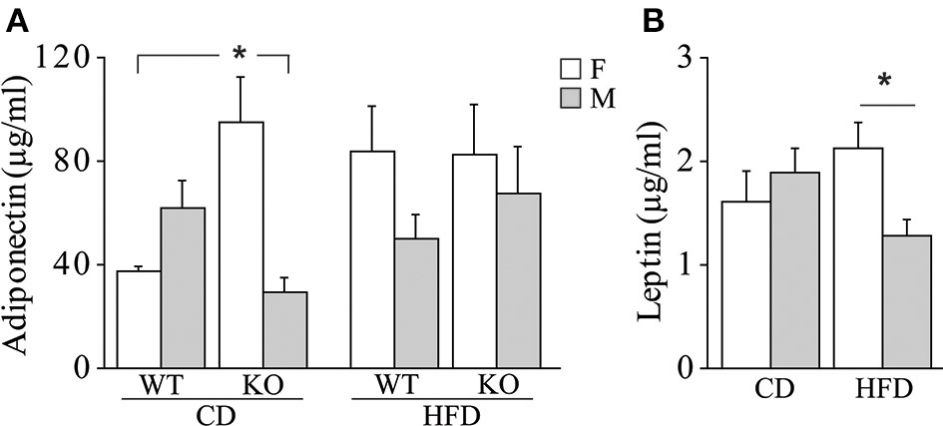
**Metabolic hormones**. Plasma levels of adiponectin **(A)** and leptin **(B)** in male and female mice prenatally exposed to CD or HFD. Data are shown as +s.e.m. *Post-hoc* comparisons, ^*^p < 0.05 (adiponectin: cd-ko-f vs. cd-wt-f and cd-ko-m; leptin: hfd-f vs. hfd-m). Experimental subjects, adiponectin assessment: cd-wt-f/m, *n* = 9; cd-ko-f/m, *n* = 8/9; hfd-wt-f/m, *n* = 8/9; hfd-ko-f/m, *n* = 8/10; leptin assessment: cd-wt-f/m, *n* = 7/9; cd-ko-f/m, *n* = 8/7; hfd-wt-f/m, *n* = 5/7; hfd-ko-f/m, *n* = 7/8.

- **Glucose tolerance test (GTT)**. In both genders and regardless of prenatal diet, p66^Shc−/−^ mice were resistant to an increase in blood glycemia upon glucose injection [main effect of genotype: *F*_(1, 77)(1, 66)_ = 11, 817; 9.018, *p* = 0.0009; 0.0038, respectively for male and female subjects, inserts Figures 6A,B]. In addition, the HFD affected the metabolic response to GTT in an opposite manner in the two genders, overriding the p66^Shc−/−^ resilience, rendering the glycemic response of p66^Shc−/−^ male mice comparable to that observed in control subjects [interaction among maternal diet, genotype and time course: *F*_(4, 308)_ = 1.078; *p* = 0.3676, Figure 6A] and, on the contrary, exacerbating the metabolic resilience of p66^Shc−/−^ females [interaction among maternal diet, genotype and time course: *F*_(4, 264)_ = 4.240; *p* = 0.0024, Figure 6B].
- **Insulin sensitivity test (IST)**. Regardless of genotype, prenatal exposure to HFD led to insulin insensitivity later in life (3-months-old) as demonstrated by the higher glycemia observed in young adults [main effect of maternal diet: *F*_(1, 77)(1, 66)_ = 3.702; 15.504, *p* = 0.05; 0.0002, respectively for male and female subjects, inserts Figures 6C,D]. As already observed in the GTT, overall, KO mice were characterized by a resilient metabolic profile showing higher blood glucose levels in response to a stimulus of insulin [main effect of genotype: *F*_(1, 77)(1, 66)_ = 21.473; 6.635, *p* < 0.0001; 0.0122, respectively for male and female subjects, inserts Figures 6C,D].
- **Metabolic hormones**. Overall, exposure to maternal HFD did not affect plasma levels of adiponectin in adult offspring [main effect of maternal diet: *F*_(1, 62)_ = 2.392; *p* = 0.1271, data not shown]. In addition, females were characterized by higher peripheral levels of adiponectin than males [main effect of gender: *F*_(1, 62)_ = 5.497; *p* = 0.0223, data not shown]. While no differences were observed among male mice regarding both diet and genotype, a greater metabolic variability was found in females. KO female mice prenatally exposed to CD were characterized by higher plasma levels of adiponectin than the WT females and KO males exposed to the same maternal diet [interaction among maternal diet, genotype and gender: *F*_(1, 61)_ = 12.691; *p* = 0.0007, Figure 7A]. Females were characterized by higher levels of adiponectin also in the adipose tissue [main effect of gender: *F*_(1, 32)_ = 6.814; *p* = 0.0136, data not shown], in particular in the mesenteric fat tissue [interaction between gender and adipose tissue: *F*_(1, 32)_ = 10.396; *p* = 0.0029, data not shown]. In addition, we found a strong tendency in increased levels of adiponectin in the mesenteric fat tissue in KO females [interaction among genotype, gender, and adipose tissue: *F*_(1, 32)_ = 3.778; *p* = 0.0608, data not shown].

Regardless of genotype, prenatal exposure to HFD led to a significantly higher plasma levels of leptin in females, compared to males [interaction between maternal diet and gender: *F*_(1, 50)_ = 5.503; *p* = 0.0230, Figure 7B].

### Neuroendocrine activation

**Figure 8:**
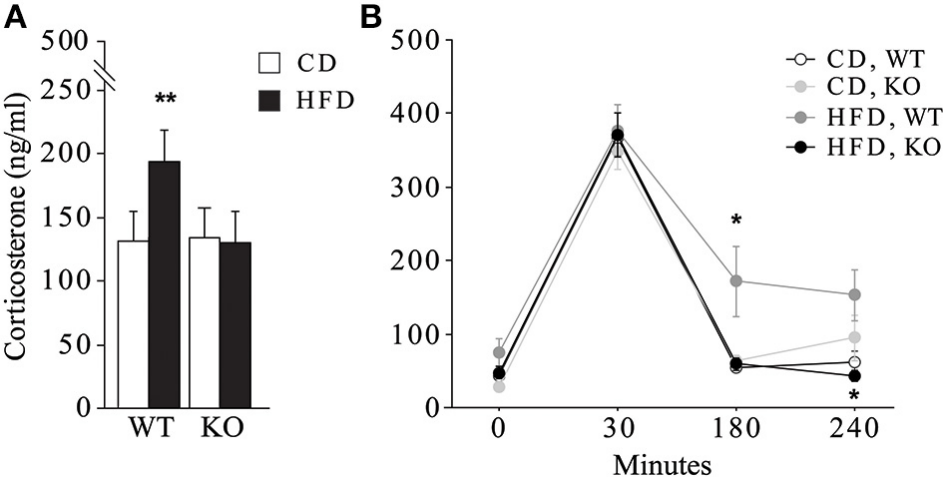
**Neuroendocrine activation**. Neuroendocrine activation in female mice in response to an acute restraint stress **(A)** and feedback response of the HPA axis activation **(B)**. Data are shown as +s.e.m. *Post-hoc* comparisons, ^*^p < 0.05 (hfd-wt vs. hfd-ko and wt-cd, at 180 min; hfd-ko vs. hfd-wt, at 240 min); ^**^p < 0.01 (hfd-wt vs. cd-wt and hfd-ko). Experimental subjects: cd-wt, *n* = 10; cd-ko, *n* = 8; hfd-wt/ko, *n* = 9.

In female mice maternal HFD exacerbated the HPA axis response to an acute psychophysical stress, increasing the plasma levels of corticosterone [main effect of maternal diet: *F*_(1, 32)_ = 6.058, *p* = 0.0194, data not shown]. The interaction between maternal diet and genotype was statistically significant [*F*_(1, 32)_ = 7.395, *p* = 0.0105, Figure 8]. In particular, while the WT females showed a marked increase in corticosterone levels in response to stress when exposed to maternal HFD, KO females were protected from the negative effects of the maternal HFD, showing a neuroendocrine response similar to KO-CD (Figure 8A). Moreover, KO females prenatally exposed to HFD showed a prompt feedback of the HPA axis activation displaying a neuroendocrine profile more similar to that of WT-CD subjects [interaction between maternal diet, genotype and time course: *F*_(3, 96)_ = 2.583; *p* = 0.05, Figure 8B]. No differences in the neuroendocrine activation were observed in male mice in response to the acute restraint stress [interaction among maternal diet, genotype and time course: *F*_(3, 87)_ = 0.735; *p* = 0.5342, data not shown].

### Behavioral phenotype

#### Open field (OF)

**Figure 9:**
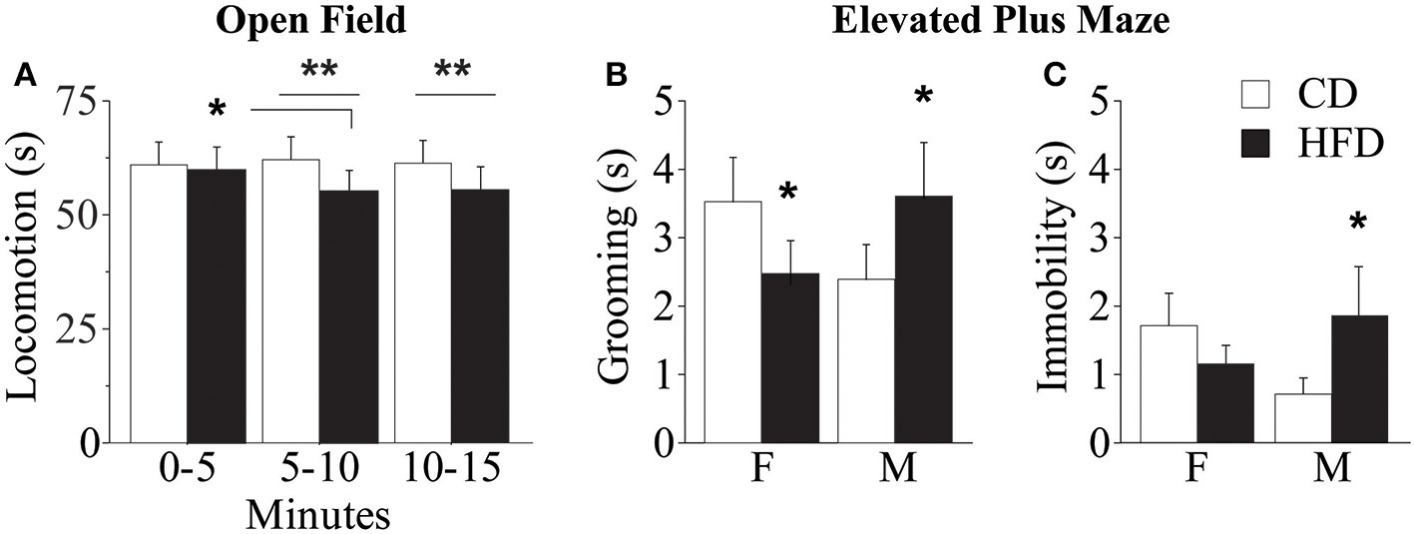
**Behavioral phenotype**. Duration of locomotor activity in the Open Field test **(A)**. Feminization of males' emotional profile prenatally exposed to HFD measured by the duration of *grooming* **(B)** and *immobility* **(C)** in the Elevated Plus Maze test. Data are shown as +s.e.m. *Post-hoc* comparisons, ^*^p < 0.05 (OF: hfd 0–5 vs. 5–10); ^**^p < 0.01 (hfd vs. cd at 5–10 and 10–15 min); ^*^p < 0.05 (EPM: hfd-f vs. cd-f; hfd-m vs. cd-m). Experimental subjects, *n* = 12.

Overall, regardless of prenatal diet and genotype, all subjects—both males and females—spent significantly less time in the center of the arena, that represents the anxiogenic portion of the apparatus [main effect of zones: *F*_(1, 56)_ = 5751.755; *p* < 0.0001, data not shown]. Regardless of both genotype and gender, prenatal exposure to HFD gradually reduced locomotion (duration of *crossings*) during the test [interaction between maternal diet and tb: *F*_(2, 178)_ = 3.910; *p* = 0.0218, Figure 9A]. While no difference emerged between HFD subjects, interestingly, from the assessment of spontaneous behavior emerged that WT subjects performed an uncommon behavior of *jumping* toward the wall of the apparatus and more frequently than KO (Fisher's exact probability test: *p* = 0.0361, data not shown). This behavior indicates an attempt to leave the apparatus and greater risk-taking and aggressive exploration representing an escaping strategy from a novel context. This data suggests that prenatal exposure to HFD reduced activity both in WT and KO mice and that overall KO subjects were less anxious than WT.

#### Elevated plus maze (EPM)

Regardless of prenatal diet, genotype and gender, overall, all subjects spent more time in the closed arms of the maze compared to both center and open arms [main effect of zones: *F*_(2, 176)_ = 29.312; *p* < 0.0001, data not shown]. In addition, prenatal diet, genotype and gender did not affect the percent frequency of entries in the open arms [main effects respectively of prenatal diet, genotype and gender: *F*_(1, 58)_ = 0.038; 0.497; 1.150; *p* = 0.8463; 0.4838; 0.2879, data not shown]. Regardless of genotype, exposure to maternal HFD *in utero* increased the duration of *immobility*, a reliable index of anxiety-like behavior, in male mice and seemed to reduce that in females [interaction between maternal diet and gender: *F*_(1, 88)_ = 3.735; *p* = 0.05, Figure 9C] suggesting a feminization of males exposed to maternal HFD. A similar gender inversion of the behavioral phenotype in response to prenatal exposure to HFD was also related to the time spent in displacement behavior (*grooming*) [interaction between maternal diet and gender: *F*_(1, 88)_ = 7.245; *p* = 0.0085, Figure 9B]. The inversion in the anxious profile in the subjects prenatally exposed to an obesogenic diet suggests that HFD affected the behavioral profile differently in the two genders, increasing anxiety in male mice and rendering females more uninhibited.

## Discussion

Overall, results of this study reveal that HFD, administered before and during pregnancy, has detrimental effects on both the dam and the offspring. Reproductive success in both WT and KO females is compromised by exposure to HFD, which leads to increased maternal mortality rate during pregnancy and increased frequency of cannibalistic episodes immediately after delivery. The most relevant findings are the long-lasting and gender-dependent effects of maternal HFD feeding on metabolic and neuroendocrine profile of the adult offspring. Maternal HFD produced more marked consequences in the adult female offspring, such as increased BMI and higher leptin levels compared to the CD group. Even greater effects were found in interaction with p66^Shc^ deletion, KO female offspring showing enhanced glucose tolerance and insulin resistance, in addition to being protected from HPA axis hyperactivity induced by HFD.

Our data indicate that high-fat feeding for 13 weeks till delivery results in maternal body weight gain accompanied by a reduced daily HFD consumption, possibly due to the high energetic and caloric intake associated with HFD. In addition, we found an increased mortality rate of pups born from HFD-fed dams, as a result of cannibalistic events and a strong association between HFD and maternal mortality in the days immediately before the expected delivery date, or during parturition. Furthermore, we found that maternal HFD results in a decreased number of pups delivered that may be due to composition of dietary fatty acids which reportedly affect fertility and induce a higher resorption number (Buckley et al., 2005). It is worth noting that similar findings have been previously reported by Flint and co-workers, in mice, and others in a different species, the rat, further corroborating the critical role played by maternal diet in such time window (Siemelink et al., 2002; Buckley et al., 2005; Flint et al., 2005). In this context, the lack of the p66^Shc^ gene plays a protective role, at least for selected parameters such as dam mortality and cannibalistic behavior.

We have observed that pups of HFD-fed dams showed a significantly lower body weight at birth (P3) but an increased weight during adolescence and at adulthood (P30 and P90). Such effect was significantly marked only in the p66^Shc−/−^ mice. These data are consistent with previous studies reporting that poor fetal growth programs pups to quickly catch-up in adult life (Ozanne and Hales, 2004; Bieswal et al., 2006). Also Flint et al. (2005) found a markedly decreased weight gain in mice pups of HFD-fed dams during the first day of lactation, as a result of an impairment of the mammary gland function, and consequent reduced milk production, induced by the diet. However, in line with our results showing an increased body weight in HFD offspring during adolescence and adulthood, such impairment was recovered in the following days (Flint et al., 2005).

Despite HFD induced differences in body weight, no change has been registered in the BMI. Such discrepancy may be due to the fact that, notwithstanding BMI is considered an adequate index of adiposity, it is poor at discriminating the ratio of fat to lean tissue (Wells et al., 2006), thus resulting not strictly related to body weight. Indeed, in the maternal-HFD exposed offspring, adult females, compared to males, had a lower body weight while showing an increased BMI.

In the present study, we did not find an effect of HFD on the onset of puberty either in males or in females. These results are apparently not in line with some previous studies showing that HFD exposure during pregnancy and/or the early postnatal period advances puberty onset in female rats (Boukouvalas et al., 2008, 2010; Chang et al., 2008; Sloboda et al., 2009; Akamine et al., 2010; Li et al., 2012). Such discrepancy may be due to the fact that percent of fat content has been suggested to be critical for the quality of the effects produced by the diet (Chang et al., 2008; Moral et al., 2011; Lie et al., 2013). Indeed, advancement of puberty onset has been found in studies using a diet containing not more than 45% of fat, while the HFD used in the current study, with a 58% fat content, did not advance puberty onset as in other studies using similar diets (Lie et al., 2013).

In addition to diet, body weight affects puberty according to an inverted relation, with increased body weight associated with earlier pubertal onset (Rosenfield et al., 2009). Accordingly, we found such inverted relation in experimental groups exposed to CD. However, in the HFD-KO group, the increased body weight did not go with a change in the mean age of puberty. This may be due to the disrupting effect of HFD on such relationship described here and in previous studies. In particular, perturbations of the timing of female puberty has been found in rats exposed to metabolic stress *in utero*, induced through administration of hypercaloric diet to the mother (Ibanez and De Zegher, 2006a,b; Ibanez et al., 2006). This effect might be mediated by an alteration of gonadal hormones secretion (Mah and Wittert, 2010; Teerds et al., 2011; Pinilla et al., 2012) resulting in a disorganized pubertal development of the offspring. Such effect warrants to be further investigated.

Here we found that p66^Shc^ gene regulates response to metabolic stimuli. In particular, KO mice show a greater resistance toward glucose challenge compared to WT. Such effect was affected by the diet in a gender-dependent fashion. Indeed, in KO females prenatally exposed to HFD, we observed a higher glucose tolerance demonstrating a better ability to metabolize the excess of blood glucose while, in KO males, we found an opposite profile as HFD determined severe glucose intolerance in comparison with their own controls (CD-KO). It is not possible to exclude that measuring glycemia sooner after the metabolic challenge (e.g., after 15 min) might provide additional information.

In the light of these points, it is reasonable to suggest that HFD overrides the effects of p66^Shc−/−^ on metabolism in males, reducing the glucose resilience, and exacerbating it in females. Accordingly, we found that the lack of the p66^Shc^ gene leads to greater resistance in the IST test. Resilience to an insulin challenge was affected not only by the genotype, but also by the maternal diet, since HFD individuals showed greater insulin insensitivity. In particular, such insulin resistance has been suggested by Turdi et al. (2013) to result from a severely dampened insulin-signaling-cascade. The metabolic alterations described in KO mice may be due to the reported involvement of p66^Shc^ gene in insulin signaling (Berniakovich et al., 2008), which leads to hypothesize that its deletion results in insulin desensitization with the consequent impairment in the metabolism of glucose.

Leptin is involved in the long-term regulation of body weight and energy balance by acting as a hunger suppressant signal to the brain and has been found to be increased in obese subjects (Hamed et al., 2011). In the present study, maternal HFD results in a difference in leptin plasma levels between genders in the adult offspring. Prior and colleagues found that offspring from HFD-fed dams exhibits an altered hypothalamic sensitivity to leptin, suggesting that metabolic alteration occurring in a susceptible time window for the developing organism impairs central mechanisms regulating leptin secretion at adulthood (Prior et al., 2013). Previous studies have already demonstrated that the maternal environment affects fetal programming, driving the development of central nervous system circuitries through a different endocrine regulation in the two genders (Maccari et al., 2003; Seckl and Holmes, 2007; Reynolds et al., 2013; Spencer, 2013).

Increased adiponectin levels were found both in plasma and in adipose tissues in KO females. Adiponectin is produced by adipocytes and its expression is inversely related to adipose tissue mass (Yamauchi et al., 2001; Antoniades et al., 2009; Ntaios et al., 2013), a result in line with the reduction of fat tissue found in KO females. In addition, as adiponectin enhances hippocampal neurogenesis (Zhang et al., 2011), the increased levels of this adipokine in KO females fit with our previous data showing increased neurogenesis in aged KO females (Berry et al., 2012).

One of the main findings of this study is the programming effect of HFD on the neuroendocrine fetal development, which leads to greater susceptibility to stressful challenges later in life. In particular, we found that HFD significantly increases the response to an acute restraint stress in adult WT females. This effect may be due to an impairment of the negative feedback of the HPA axis, as suggested by the long lasting increase in corticosterone levels induced by the stressor. Interestingly, despite their exposure to maternal HFD, KO females were protected from such effect, showing an efficient HPA axis feedback, undistinguishable from CD. It is worth noting that the alterations of the neuroendocrine profile induced by maternal HFD overlap with those induced by prenatal stress. For instance, Louvart and colleagues reported that female offspring of rat dams exposed to a chronic restraint stress during the last trimester of pregnancy display at adulthood a higher response to restraint stress (Louvart et al., 2009). The overlapping consequences of maternal HFD and exposure to stressful conditions during the prenatal period leads us to hypothesize that maternal HFD mimics psychophysical stress, as both interfere with the fetal programming of neuroendocrine development, altering the HPA axis activity during the entire life span (Maccari et al., 2003; Seckl and Holmes, 2007; Reynolds et al., 2013; Spencer, 2013).

Previous studies have reported increased anxiety-like behavior in adult subjects of both genders exposed to HFD during fetal development (Peleg-Raibstein et al., 2012), although in some cases a gender dependent effect is reported (Bilbo and Tsang, 2010). In line with the latter finding, we show different effects in males and females. In particular, we observed increased grooming levels in the EPM, considered an endpoint of anxiety-like behavior, in CD females compared to CD males. Such difference in basal levels of anxiety response between genders is widely described in the literature (Kessler et al., 1994, 1995; Weissman et al., 1994, 1996; Gater et al., 1998). However, such profile was inverted by the exposure to maternal HFD, suggesting a feminization of the emotional profile of male mice. This is especially interesting in light of the results from prenatally stressed subjects, which are characterized by the same feminization effect (Weinstock, 2001). Such finding corroborates the idea that prenatal metabolic alterations acts as a psychophysical stressful stimulus with potential implications for brain function, both at adulthood and during the aging process, confirming the data about the neuroendocrine profile observed in HFD-WT females (Maccari et al., 1995, 2003; Spencer, 2013).

Overall, results from this study indicate that maternal HFD might represent a detrimental metabolic stimulus for the dam and, in particular, for the offspring. Since our model of HFD feeding did not lead to maternal obesity, the observed effects on the offspring are purely due to alterations of maternal metabolism. In particular, in our study, maternal HFD seems to be a condition sufficient to induce “metabolic misprogramming” causing offspring metabolic and neuroendocrine alterations. Moreover, the gender-dependent effects here described might suggest a potential modulating role played by sex hormones on the long-term consequences of maternal HFD on the offspring. To explore such hypothesis further studies are warranted.

Finally, the overall resilience of the KO in response to metabolic challenges confirms the notion that p66^Shc^ is an important molecular target for future studies investigating pathological states induced by stressful or metabolic factors during early life.

### Conflict of interest statement

The authors declare that the research was conducted in the absence of any commercial or financial relationships that could be construed as a potential conflict of interest.
